# A global flash drought inventory based on soil moisture volatility

**DOI:** 10.1038/s41597-024-03809-9

**Published:** 2024-09-04

**Authors:** Mahmoud Osman, Benjamin Zaitchik, Jason Otkin, Martha Anderson

**Affiliations:** 1 Department of Earth and Planetary Sciences, Johns Hopkins University, Baltimore, MD USA; 2 Irrigation and Hydraulics Department, Cairo University, Cairo, Egypt; 3 Space Science and Engineering Center, Cooperative Institute for Meteorological Satellite Studies, University of Wisconsin–Madison, Madison, WI USA; 4 Hydrology and Remote Sensing Laboratory, Agricultural Research Service, USDA, Maryland, MD USA

**Keywords:** Hydrology, Natural hazards

## Abstract

Flash droughts, characterized by rapid onset and development, present significant challenges to agriculture and climate mitigation strategies. Operational drought monitoring systems, based on precipitation, soil moisture deficits, or temperature anomalies, often fall short in timely detection of these events, underscoring the need for customized identification and monitoring indices that account for the rapidity of flash drought onset. Recognizing this need, this paper introduces a global flash drought inventory from 1990 to 2021 derived using the Soil Moisture Volatility Index (SMVI). Our work expands the application of the SMVI methodology, previously focused on the United States, to a global scale, providing a tool for understanding and predicting these rapidly developing phenomena. The dataset encompasses detailed event characteristics, including onset, duration, and severity, across diverse climate zones. By integrating atmospheric variables through their impact on soil moisture, the inventory offers a platform for analyzing the drivers and impacts of flash droughts, and serves as a large, consistent dataset for use in training and evaluating flash drought prediction models.

## Background & Summary

The phenomenon of rapid onset “flash drought” events has gained significant attention in the past decade due to their sudden impact on ecosystems, agriculture, and water resources. These droughts can develop within weeks, leaving little time for effective mitigation and response^1–3^. Traditional drought indices often fail to capture the swift nature and immediate impacts of these events, leading to a gap in effective monitoring and prediction. To address this gap, recent studies have focused on developing indices designed to capture the characteristic rapid development of flash droughts to inventory and map these events. Otkin *et al*.^4–6^ identified flash droughts based on rapid changes in the ratio between actual evapotranspiration (EVP) and potential evapotranspiration (PEVP). Other researchers, such as Hunt *et al*.^7^ and Mo and Lettenmaier^8,9^, defined flash droughts by the rapid decline in soil moisture over time. Chen *et al*.^10^ suggested that the onset of flash droughts could be defined by a two-category degradation in the U.S. Drought Monitor (USDM) within four weeks. Christian *et al*.^11^ introduced a definition based on the rate of change in the standardized ratio between EVP and PEVP over a six-pentad period, while Ford and Labosier^12,13^ identified flash droughts as a drop in pentad-averaged soil moisture from the 40^th^ to the 20^th^ percentiles within four pentads or less. Hoffmann *et al*.^14^ refined this methodology to reduce the number of identified events. Osman *et al*.^3^ proposed a definition based on the Soil Moisture Volatility Index (SMVI) and compared it with six other definitions to emphasize the variety of pathways for identifying flash drought onset.

While many of these studies have focused on the contiguous United States (CONUS), flash droughts have been observed globally, including in China and India, as noted by studies such as Wang *et al*.^15^, Yuan *et al*.^16^, and Mahto and Mishra^17^. These international studies have contributed additional definitions, highlighting the need to understand the implications of different flash drought definitions, a research question in its own right (Lisonbee *et al*.^2^). Efforts to quantify the severity of flash droughts are less common but informative. Chen *et al*.^10^ and Otkin *et al*.^6^ used USDM categories to diagnose and assess flash drought severity, while Christian *et al*.^11^ employed the standardized evaporative stress ratio (SESR). Yuan *et al*.^16^ used soil moisture deficit, and Li *et al*.^18^ used evapotranspiration deficit. Otkin *et al*.^19^ developed a flash drought intensity index (FDII) based on modeled soil moisture, accounting for both the rapid intensification magnitude and resultant drought severity. Their study revealed significant regional differences in flash drought severity when both components were considered. Most definitions and intensity metrics for flash droughts focus on capturing the phenomenon rather than assessing it as a coherent class of drought processes. An exception is Mo and Lettenmaier^8,9^, who distinguished between precipitation deficit flash droughts and heat wave flash droughts, although their approach has been debated due to their focus on heatwave duration rather than intensification rate, which is typically seen as the defining characteristic of flash droughts^1,2^.

The SMVI, introduced by Osman *et al*.^3,20^ in studies of flash droughts in the United States diagnoses flash drought conditions by identifying events in which the short-term decline in soil moisture is particularly severe relative to more gradual trends. In using a rate-based metric to define flash droughts, the SMVI is consistent with several other flash drought indices proposed in recent years^2^. SMVI applications to the United States have proved to be useful for capturing flash drought events and for supporting classification and prediction studies^3,20,21^, and the method has begun to be applied in other regions^22^. Ford *et al*.^21^ used SMVI in an intercomparison of nine flash drought indicators across the contiguous United States, finding that while no single indicator consistently outperformed others, SMVI was valuable as part of a multi-indicator approach due to its ability to capture soil moisture dynamics. Osman *et al*.^20^ utilized SMVI to classify flash droughts into distinct types based on meteorological and land surface conditions, demonstrating its effectiveness in diagnosing rapid soil moisture changes and identifying different flash drought classes. Alencar and Paton^22^ compared six flash drought identification methods in Central Europe, including SMVI used as a reference method, and found it particularly suited for identifying rapid soil moisture declines in croplands, but noted the variability in identified drought periods due to different definitions. These studies collectively suggest that SMVI effectively captures rapid soil moisture changes crucial for detecting flash droughts, with its optimal use as part of an ensemble approach with other indicators to account for the complexity and variability of flash drought events.

The dataset introduced here leverages the SMVI methodology to create a consistent global flash drought inventory for the period 1990 to 2021. This work aligns with the growing need for accurate, timely drought monitoring tools in the context of climate change, as highlighted in numerous studies (e.g^1,4–19,22–30^). By offering a consistent, scalable approach, this dataset can contribute to the development of flash drought forecasts and resilience strategies, addressing a critical need in climate resilience and climate change adaptation efforts.

The application of SMVI at global scale presents challenges and opportunities beyond those encountered in regional-scale studies. The remainder of this paper describes the approach used to apply SMVI globally and describes key characteristics of the dataset.

## Methods

The development of the SMVI Global Inventory involved a systematic approach to expand the application of the SMVI methodology, originally tailored for the United States, to a global scale. Our methodology began with an adaptation of the SMVI, a tool designed to identify and quantify flash drought events through the volatility of a single-variable approach. This adaptation was informed by the studies of Osman *et al*.^3,20^, which provided a robust framework for flash drought detection. To tailor SMVI to global use, we modified the original criteria used in these studies, accounting for regional variations in climate and environmental conditions, as well as our up-to-date understanding of flash droughts onset and impacts.

**Fig. 1:**
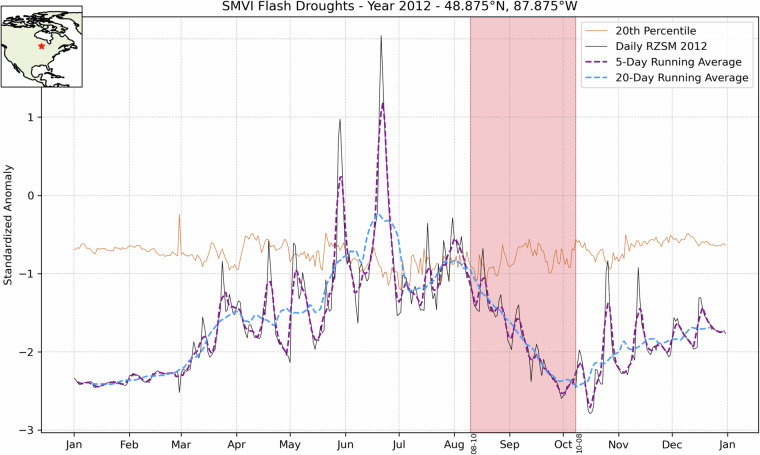
SMVI definition applied to a grid-point at 48.875 N, 87.875 W within the United States’ Midwest region in 2012 revealing a flash drought event, indicated by the shaded red region.

For the global inventory, flash droughts are identified based on a set of criteria that are derived but adjusted from those presented for the United States in Osman *et al*.^3^ (and repeated here in Supplementary Material, for reference). As depicted in the example in Fig. 1, the criteria are:

1. A decrease in the 5-day running average (pentad) of Root Zone Soil Moisture (RZSM) below the 20-day running average. This criterion captures rapid changes in soil moisture, a key indicator of flash drought onset.
2. The occurrence of this decline below the 20^th^ percentile RZSM based on the long-term record for the corresponding day, signifying an abnormal moisture deficit. The selection of the 20^th^ percentile threshold was based on recommendations from the US Drought Monitor (USDM), which identifies it as indicative of “Moderate Drought – D1” conditions.
3. The persistence of these conditions for a minimum of four pentads, confirming that the initial drying lasts long enough to have meaningful impacts.
4. An adjustment to the originally presented SMVI (Osman *et al*.)^3^: An average temperature threshold of above 0 °C and a specific range in Bowen ratio (B, defined as the ratio of sensible to latent heating) of 0.2 to 7 during the drought period. These additional constraints ensure that droughts are only identified under relevant meteorological conditions, and avoid identifying false events due to rapid changes in soil moisture in desert regions (very high values of B) or heavily forested lands (very low B).

In previously presented criteria, we conducted tests with different moving average windows, including the 20-day window recommended by experts from the USDA and The National Drought Mitigation Center. These tests demonstrated that while various window sizes yielded comparable results, the 20-day moving average effectively smoothed out short-term fluctuations in soil moisture data, enhancing our ability to discern longer-term trends and variations. We have explained the methodology in more details in Osman *et al*.^3,20^ including comparison with different flash drought identification methods.

Once a flash drought is identified in a grid point, its severity is quantified based on the RZSM deficit, adhering to Eqs. 1 and 2 derived from Osman *et al*.^20^. In this formulation, severity is calculated as the difference between the 5-day running average RZSM and the 20^th^ percentile RZSM for that location at that time of year.

1 $${SV}=\mathop{\sum }\limits_{t={t}_{o}}^{t={t}_{f}}({RZS}{M}_{20{th}}-{RZS}{M}_{5d})$$

2 $$S{V}_{{Cat}}={STD}\left(S{V}_{1990-2021}\right)$$

Where SV is the computed severity, and ${RZS}{M}_{20{th}}$ and ${RZS}{M}_{5d}$ are the 20^th^ percentile and 5-day moving average RZSM, respectively. Parameters ${t}_{o}$ and ${t}_{f}$ represent the times at which identified flash drought onset occurs and recovers, respectively. $S{V}_{{Cat}}$ represents the standardized severity category calculated from the flash drought inventory for all grid points, measured against the severity of all other identified flash drought events within the inventory.

In Fig. 1, though the RZSM for the selected grid point is observed below the 20^th^ percentile climatological RZSM for an extended period, a flash drought event cannot be identified as the RZSM is already rising. The rapid drop in RZSM starts later in the summer (~10^th^ of August) and keeps dropping fast enough for at least four pentads till it starts a recovery around the 8^th^ of October. The period between the 10^th^ of August and 8^th^ of October marks a flash drought event with a severity equivalent to the integration of the soil moisture deficit that lies in the figure between the 20^th^ percentile and the 5-day running average.

This quantitative assessment allows for a consistent and objective evaluation of drought severity across different regions. The end of a flash drought event (“recovery”) is defined when the rate of drop in RZSM during an identified SMVI flash drought event begins to recover, i.e. SMVI criteria are violated.

Finally, if two (or more) flash drought events are identified in the same grid cell with onset dates that are three pentads or less apart, they are considered as one combined event starting on the date of the first event’s onset and recovering on the date of the last event’s conclusion. This is based on the practical understanding that a brief wetting period will not be enough to relieve the first flash drought event if it is rapidly followed by another event.

Multiple events are permitted to be detected for each grid cell in each calendar year. The inventory sets a threshold of six events, as higher frequencies were not observed.

All SMVI calculations use the root zone soil moisture (RZSM) field (0–100 cm below surface) from the NASA Global Land Data Assimilation System Version 2 (GLDAS-2) using the Catchment Land Surface Model (CLSM) version 2.5^31–33^. This dataset provides a comprehensive and continuous daily record of RZSM at 0.25-degree horizontal resolution, offering an extensive temporal scope for analyzing environmental and climatic root zone soil moisture trends from 1980 to 2021. The selection for GLDAS is based on its ability to provide enhanced hydrological representation by incorporating GRACE data assimilation, that is expected to uniquely captures the complex interactions between soil moisture and groundwater, offering a more comprehensive understanding of the water balance^31–33^. GLDAS-2 standardized RZSM anomalies are included in the SMVI Global Inventory to provide context for the diagnosed flash drought events. Low RZSM values are indicative of dry conditions that can stress natural vegetation and crops, as RZSM provides insights into the capacity of the soil to support plant life and the potential for agricultural impacts, which are key concerns during flash drought conditions.

In addition, the SMVI Global Inventory includes a suite of standardized anomalies of meteorological variables aligned with the flash drought inventory. These variables are provided so that users of the inventory can contextualize events and understand the meteorological factors driving flash drought. This integration is vital for a comprehensive analysis of flash drought dynamics and their associated meteorological conditions.

The meteorological variables integrated into the dataset include Total Liquid Precipitation (ARAIN), Actual Evapotranspiration (EVP), Surface Pressure (PRESS), 2-m above ground Temperature (TMP), Vapor Pressure Deficit (VPD), and 10-m above ground Wind Speed (WS), along with Potential Evapotranspiration (PEVPR). These variables were selected for their relevance in characterizing the surface and atmospheric state during flash drought occurrences. The particular version of GLDAS used in our inventory did not include all of the relevant meteorological variables, so these fields were sourced from the ERA5 reanalysis from the European Centre for Medium-Range Weather Forecasts (ECMWF). ERA5 is part of the Copernicus Climate Change Service (C3S) and represents one of the most advanced atmospheric reanalyses available^34^.

Each of these meteorological variables offers a distinct perspective on the conditions contributing to the onset, intensification, and cessation of flash droughts. For instance:

- Total Liquid Precipitation (ARAIN) is a key meteorological variable that is crucial for understanding the moisture input into the soil system. Low ARAIN values can be a primary indicator of insufficient moisture replenishment, leading to potential soil dryness. In flash drought scenarios, a sudden drop in ARAIN, especially during typically wetter periods, can rapidly escalate drought conditions by failing to offset increased evaporation and transpiration rates.
- Actual Evapotranspiration (EVP) refers to the actual transfer of moisture from the Earth’s surface to the atmosphere, combining both evaporation from land and water surfaces and transpiration from plants. It directly indicates the rate at which moisture is being removed from the soil and vegetation. High EVP rates, particularly during periods of low precipitation (ARAIN), can significantly contribute to the rapid depletion of soil moisture. EVP is influenced by various factors, including temperature, solar radiation, humidity, wind speed, and soil moisture levels.
- Surface Pressure (PRESS) is indicative of broader weather patterns which in turn affect moisture availability and atmospheric conditions conducive to drought development.
- Temperature (TMP) – 2 m above ground level, influences evaporation and transpiration rates, with higher temperatures accelerating soil moisture loss. Analyzing temperature anomalies helps assess the thermal stress on ecosystems and agriculture, providing insights into the potential severity of flash drought impacts.
- Vapor Pressure Deficit (VPD) measures the ‘dryness’ of the air, indicating its capacity to absorb moisture, a key factor in the rapid development of flash drought conditions.
- Wind Speed (WS) influences evapotranspiration rates and moisture transport. Higher wind speeds can increase evaporation rates by moving more air over the soil and vegetation surfaces, effectively removing the moisture more rapidly. This process can lead to quicker drying of the soil.
- Potential Evapotranspiration (PEVPR) represents the maximum amount of moisture that could be evaporated and transpired from the land surface under prevailing atmospheric conditions, assuming no limitations in water availability. It provides an estimate of the atmospheric demand for moisture. High PEVPR values indicate conditions conducive to rapid soil moisture depletion, especially when actual soil moisture is low. This disparity between potential and actual evapotranspiration can signal the onset of drought conditions.

By analyzing these variables at various stages of the drought event – pre-onset, onset, drought, and recovery – one can gain a detailed understanding of the atmospheric anomalies associated with each phase of a flash drought.

The SMVI global flash droughts dataset inventory currently covers the period from 1990 to 2021 offered with a spatial resolution of 0.25 degrees, similar to that of GLDAS, providing a satisfactory level of detail for both global and regional climate studies.

## Data Records

The SMVI Global Flash Drought Inventory dataset comprises a comprehensive collection of flash drought events identified globally from 1990 to 2021. This inventory is hosted on HydroShare^35^, providing open access to the research community for further analysis and application. The dataset is organized into two sub-directories: 1- “FD_Events”, 2-“Composites”, and one single “.csv file” (LonLat.csv). All files are stored in a common data format (CSV) suitable for handling multidimensional arrays of scientific data with low storage requirements. The following is the structure of the dataset’s directory:

1. FD_Events: This directory catalogs detected flash drought events as described in (Osman *et al*.^3^), one file per year with the following header format:
  fstdate#: Date of onset of event #n
  lstdate#: Date of recovery (end of rapid intensification) of event #n
  SV#: Severity of event #n
  VEGID: Landcover class number according to GLDAS dominant vegetation type in CLSMF2.5^31–33^.
2. Composites: This section includes atmospheric standardized anomalies associated with flash drought events at various stages (onset, pre-set, and recovery). Separate files are designated for each variable per year and event, formatted as SMVI_GLDAS_E[event#]_[variable name]_[Year].csv, covering the following variables:
  ARAIN: Total Liquid Precipitation
  EVP: Actual evapotranspiration
  PRES: Surface pressure
  RZSM: Root-zone soil moisture
  TMP: 2-m above ground temperature
  VPD: vapor pressure deficit
  WS: 10-m above ground wind speed
  PEVPR: potential evapotranspiration
3. “LonLat.csv This file enumerates the longitudinal and latitudinal coordinates corresponding to each row across the dataset, essential for geographic referencing of the flash drought events.

For user convenience, each of the FD_Events and Composites directories includes a NetCDF directory, allowing users to choose their preferred method of use.

The dataset is designed for ease of access and usability, providing a foundation for significant advancements in understanding, predicting, and managing flash drought phenomena globally. The dataset, including its comprehensive structure and the associated README file detailing its use, is available at the HydroShare repository^35^ along with a sample R script to guide users through the initial steps of data loading and visualization.

## Technical Validation

The data used in creating the inventory is pre-processed using multiple tools, including Unix shell scripts utilizing Climate Data Operators (CDO) and NCO commands to calculate the long-term means, percentiles, and anomalies. The rest of SMVI calculations are performed in R.

The pre-processed RZSM data from GLDAS is the main component of the SMVI calculation process. All RZSM thresholds and conditions are based on the GLDAS dataset, as are additional criteria, such as the Bowen ratio requirement computed as Sensible heat net flux (Qh_tavg) divided by Latent heat net flux (Qle_tavg).

**Fig. 2:**
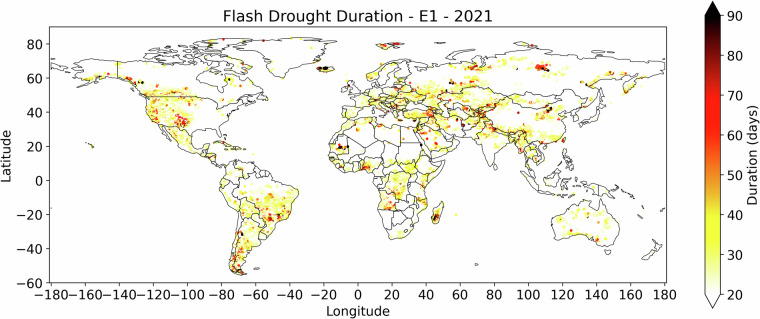
Number of days in the first identified SMVI flash drought event in year 2021.

The resulting inventory is a comprehensive dataset of all detected events in each calendar year (Jan 1^st^ to Dec 31^st^). Events are arranged based on their onset dates, for example: E1 refers to the first detected flash drought event (fulfilling the defined SMVI conditions) for a given year. Figure 2 shows an example of a map for the duration of E1 for year 2021.

**Fig. 3:**
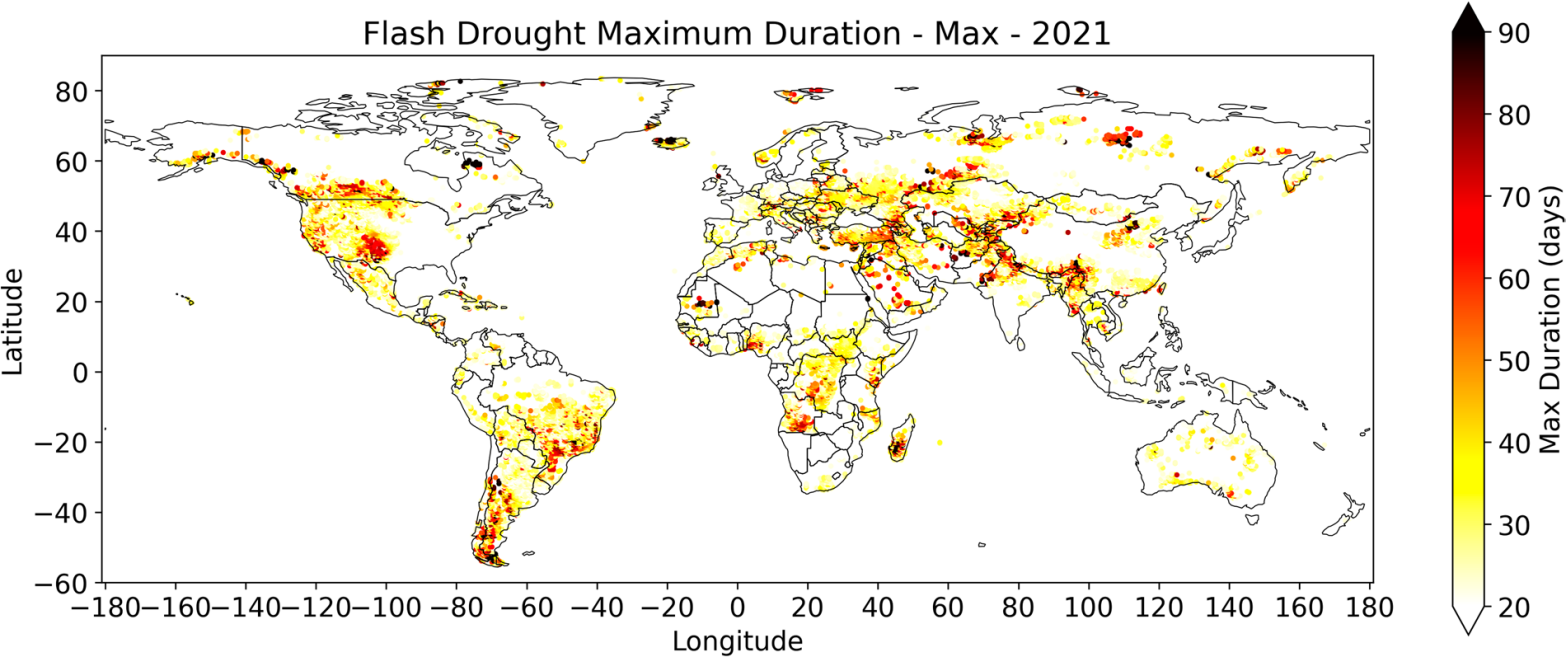
Calculated number of days of the maximum-duration identified SMVI flash drought event in year 2021.

Due to the presence of multiple detected events in the created flash droughts dataset, where each grid cell might experience different events of different lengths, the maximum duration flash drought event is not necessarily the first event, so it needs to be calculated from the dataset as the maximum number of days among all identified events for that grid cell in that year, as shown in Fig. 3.

**Fig. 4:**
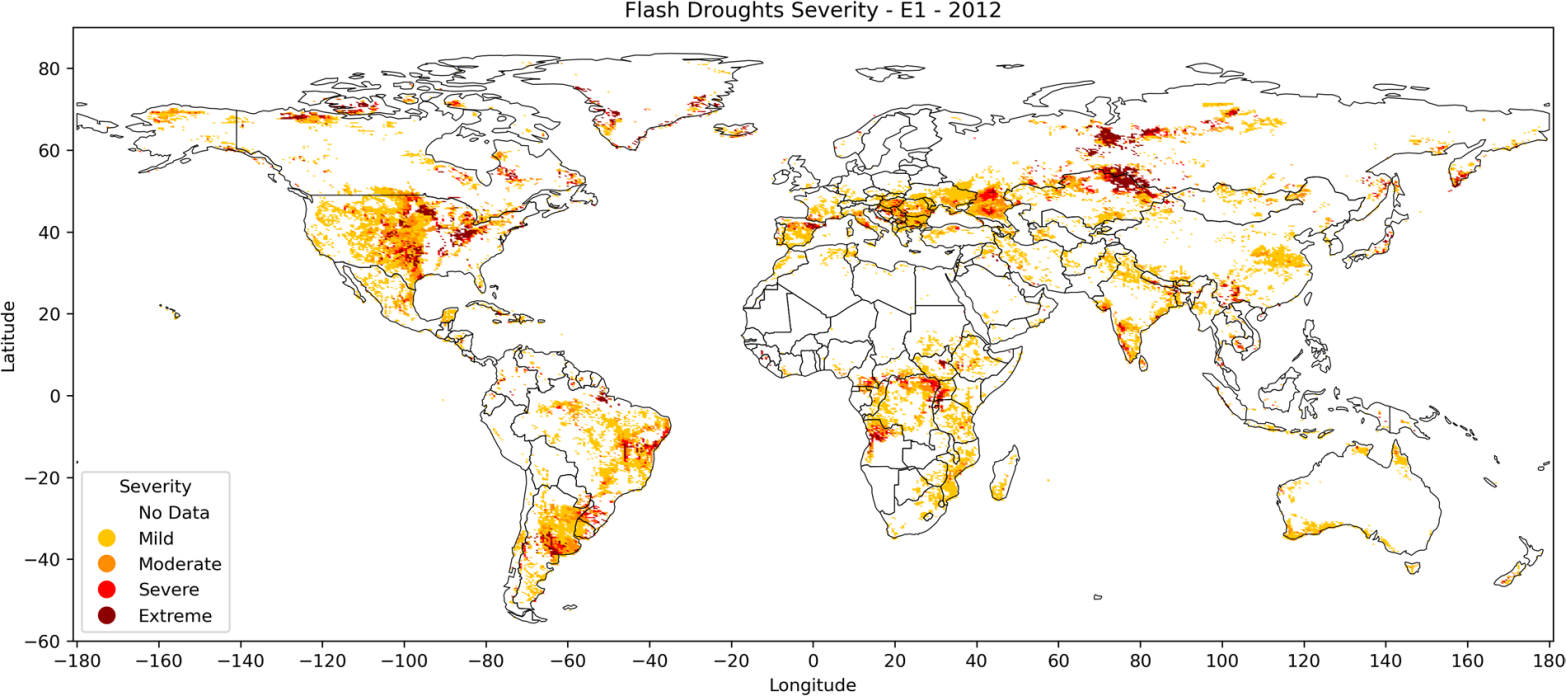
Classified severity map for the identified SMVI flash droughts for the first detected event in year 2012.

Severity, as explained in the methodology section, is represented in the dataset as the summation of the deficit in RZSM, based on the difference between the 5-days running average RZSM and the 20^th^ percentile of RZSM at that location and time of year. Severity can be depicted in multiple ways. In Fig. 4 we show severity in a categorical form based on Z scores computed from the full inventory’s global mean and standard deviation. Flash drought events are characterized as Mild for Z score less than 0, Moderate for Z score between 0 and 0.5, Severe for Z score between 0.5 and 1, and Extreme for Z score more than 1. However, in the published SMVI global dataset^35^, we leave the severity values as explained in the methodology section without defining categories’ thresholds, so that the user can set them according to the needed application.

**Fig. 5:**
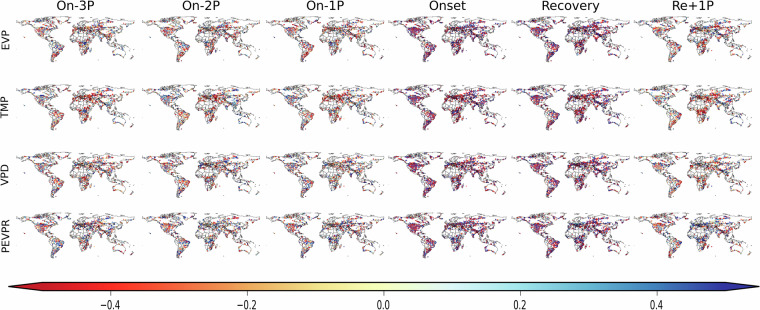
Standardized anomalies of selected fields associated with the SMVI detected flash droughts in year 2012. Note that marker points are magnified to show colors clearly.

Meteorological variables are pre-processed in a similar manner to SMVI using UNIX shell scripts and then processed in R for the computation of the anomalies at different time intervals associated with the onset, persistence, and recovery of all detected flash drought events. Timesteps are defined as the 1-pentad average anomalies of the selected field at these times: Onset (On00), 1-pentad prior to onset (On01), 2-pentads prior to onset (On02), 3-pentads prior to onset (On03), Recovery (Re00), 1-pentad after recovery (Re01) and a 3-pentads averaged computed anomalies of the selected field prior to onset (On3Pn). Figure 5 shows an example of the global anomalies of selected environmental fields associated with the first detected flash drought event (E1) in year 2012.

Since application of the inventory can vary widely for different land cover types, the inventory includes a field for the land cover class number according to GLDAS dominant vegetation type in CLSMF2.5^31–33^.

The ability of the SMVI flash drought index to capture flash drought events was evaluated in Osman *et al*.^3,20^ using a number of criteria drawn from field observations and satellite-derived vegetation conditions. Following from these results, the SMVI has been used as a stable reference method for identifying flash droughts^21^.

The SMVI-based global flash drought inventory is intended to provide a globally consistent, process-appropriate inventory of flash drought events along with meteorological anomalies relevant to their progression. The dataset is not without limitations. The SMVI calculation depends on the quality of input RZSM data and other fields, so limitations in the GLDAS representation of these fields propagate through the flash drought inventory. Future work could include an expansion of the inventory to include additional reanalysis datasets and, perhaps, multiple flash drought definitions.

Notwithstanding these limitations, the current inventory is offered as a step towards consistent and comprehensive flash drought inventory and analysis. The field of flash drought studies has grown rapidly in recent years, and there has been exciting progress in the understanding and prediction of these events. But literature has suffered to some extent from inconsistencies in flash drought datasets and definitions that can make it difficult to compare across studies from different regions or analytical frameworks. A move towards globally consistent datasets that include flash drought inventories and relevant meteorological fields can support more robust and comparable studies of this high impact class of drought.

## Supplementary information

Supplementary Material

## Data Availability

A GitHub public repository has been established to assist users in processing data. This repository will receive updates in response to feedback or contributions from users, as well as any modifications to the dataset. Access this repository via https://github.com/mosman01/SMVI. Additionally, the Global SMVI inventory, documented by Osman *et al*.^35^, is accessible to all researchers at 10.4211/hs.080002bd7cc44242bb37c02b049ed532.
