# Supplementary material for: A global flash drought inventory based on soil moisture volatility
Source: Sci Data. 2024 Sep 4;11:965. doi: 10.1038/s41597-024-03809-9 (PMC11375007; doi:10.1038/s41597-024-03809-9)
Supplement: Supplementary file 1 — Supplementary Material [file 41597_2024_3809_MOESM1_ESM.pdf]

A global flash drought inventory based on soil moisture volatility

Mahmoud Osman[1,2]; Benjamin Zaitchik[1]; Jason Otkin[3]; Martha Anderson[4]

[1] Department of Earth and Planetary Sciences, Johns Hopkins University, Baltimore, MD, USA.

[2] Irrigation and Hydraulics Department, Cairo University, Cairo, Egypt.

[3] Space Science and Engineering Center, Cooperative Institute for Meteorological Satellite Studies, University of Wisconsin–Madison, WI, USA.

[4] Hydrology and Remote Sensing Laboratory, Agricultural Research Service, USDA, MD, USA.

**Corresponding author: Mahmoud Osman[1,2] – Email address: mahosman01@gmail.com; mahmoud.osman@jhu.edu*

Supplementary Material:

The original criteria for SMVI quoted from Osman et al. 2021, section 2.1:

"As flash droughts are characterized by rapid onset, we adopt an approach inspired by studies of market volatility, where robust identification of rapid yet significant changes in stock prices is critical. In this definition, a flash drought is said to occur when: (1) the 1-pentad (5 day) running average root zone soil moisture (RZSM) falls below the 4-pentad (20 day) running average for a period of at least 4 pentads; (2) by the end of the period, RZSM drops below the 20th percentile for that time of year according to the 1979-2018 period of record."
